# Supplementary figures and images for: Impact of early detection on cancer curability: A modified Delphi panel study
Source: PLoS One. 2022 Dec 21;17(12):e0279227. doi: 10.1371/journal.pone.0279227 (PMC9770338; doi:10.1371/journal.pone.0279227)

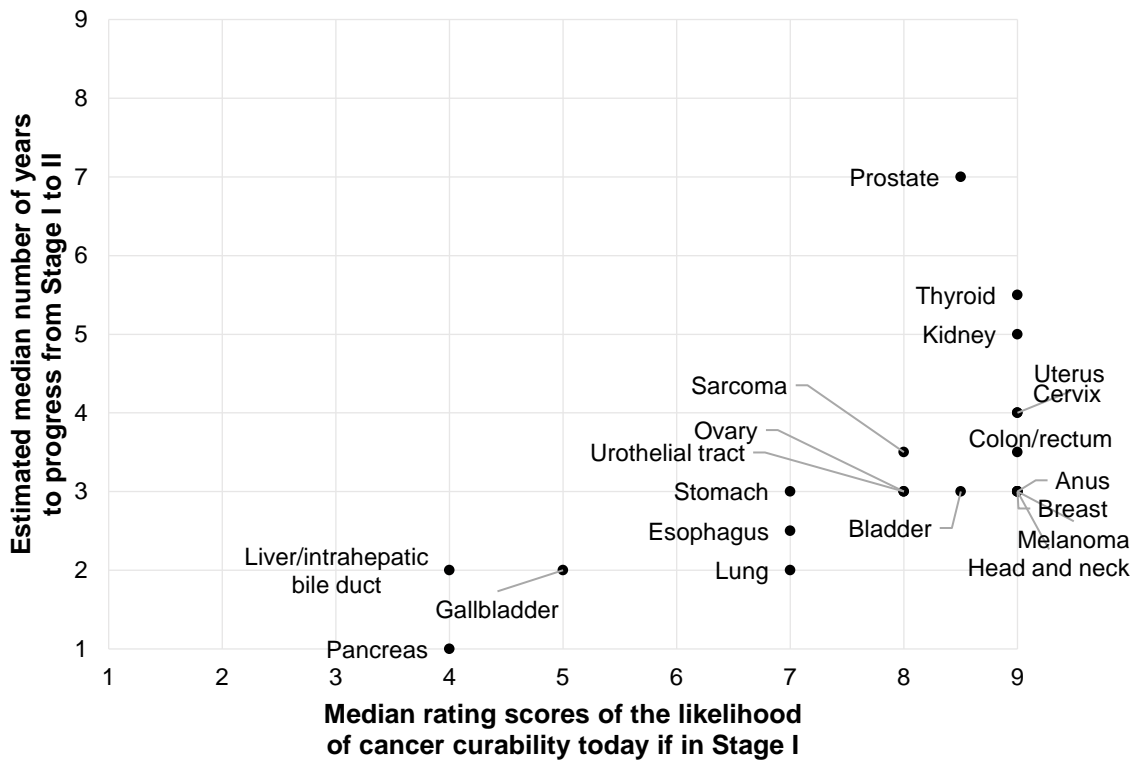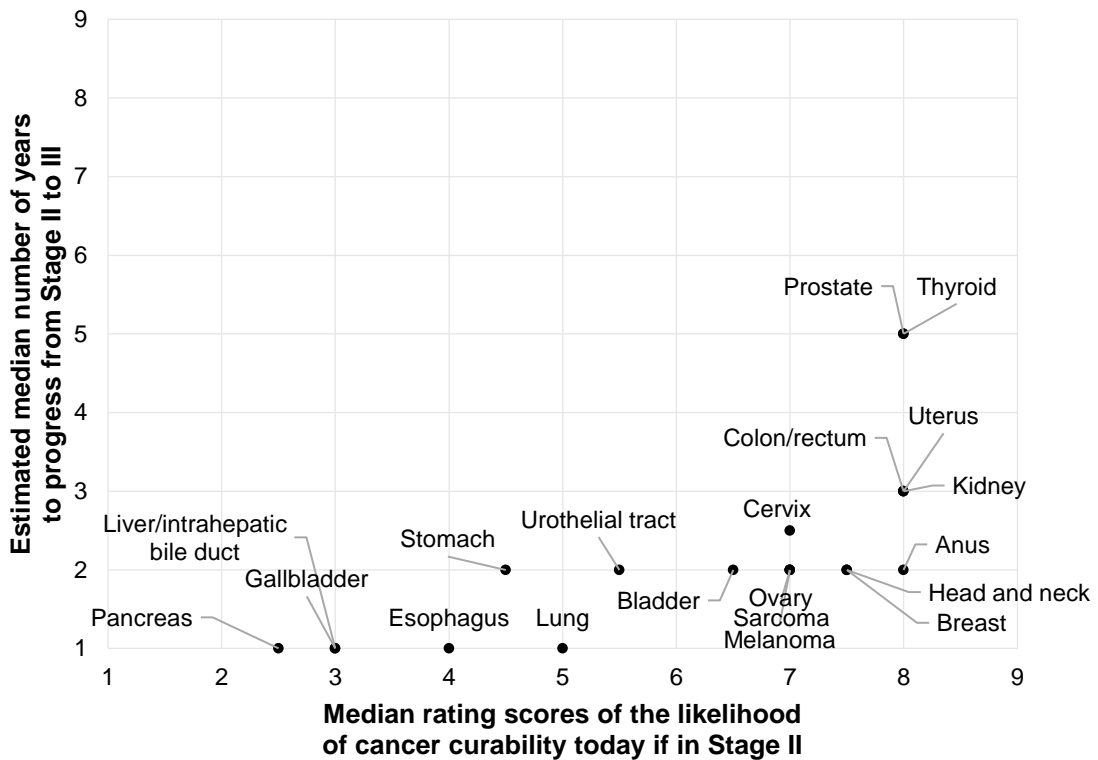

**S1 Fig. Estimated cancer curability at a given stage and progression to the next stage.**

Supplement: S1 Fig — (PDF) [file pone.0279227.s001.pdf]
