# Supplementary material for: Impact of early detection on cancer curability: A modified Delphi panel study
Source: PLoS One. 2022 Dec 21;17(12):e0279227. doi: 10.1371/journal.pone.0279227 (PMC9770338; doi:10.1371/journal.pone.0279227)
Supplement: S1 Table — (PDF) [file pone.0279227.s002.pdf]

All Panelists

Median (Range)
Yellow: Disagreement (≥2 ratings of 1-3 and ≥2 ratings of 7-9)
Blue 1: Median ≥7-9 without disagreement
Blue 2: Median ≥4-<7, without disagreement
Blue 3: Median 1-<4 without disagreement

The following document presents the group's median and range of ratings. Each cell is color coded. Cells in yellow are ones where the group disagreed (≥2 panelists gave that cell a rating of 1-3 and ≥2 panelists gave that cell a rating of 7-9); cells in blue are ones where the group agreed, with the darker blues representing higher medians, as shown in the key on the right.

SECTION 1

TABLE 1. SOLID ORGAN CANCERS

Please enter a rating in each yellow cell, where each cell represents a cancer or cancer stage. If there is heterogeneity within a category, do your best to imagine a **typical** patient.		Q1: What is the likelihood that patients diagnosed with this cancer at this stage today will be cured [1] with **typical treatment** [2]?			Q2: What is the likelihood that **untreated patients** with stage I or II of this cancer will progress to stage III or IV disease over their lifetime?			Q3: How long does it take this cancer to progress from the beginning of this stage [3] to the beginning of the next stage in undiagnosed patients (natural history of disease)?	Imagine an annual hypothetical screening blood test for patients ≥50 years of age that is 100% sensitive and 100% specific for the stage of cancer you are being asked to rate. Assume this test cannot differentiate between higher and lower risk cancers. If all cancers of this type were diagnosed no later than the stage [4] indicated by the row heading, to what extent would… Q4: …**life expectancy** increase in **all** patients with this cancer, relative to life expectancy without the screening test [6]? Assume patients are receiving **typical treatment**.	…**cure rates** increase in **all** patients with this cancer, relative to cure rates without the screening test? Q5: Assume patients are receiving **typical treatment**.	Q6: Assume patients are receiving **best available treatment** [7].	Now consider what medical practice might be like in 2030 [5] with cure rates higher than today's. Q7: If all cancers of this type were diagnosed no later than this stage, to what extent would **cure rates** increase with **typical treatment** in **all** patients with this cancer, relative to 2030 cure rates without the screening test?
		For columns A through F, enter a rating of 1 through 9, where: ***1*** *=Extremely unlikely* ***3*** *=Somewhat unlikely* ***5*** *=Neutral, neither likely nor unlikely* ***7*** *=Somewhat likely* ***9*** *=Extremely likely*						*For column G, enter a rating of 1 through 9, where:* ***1*** *=Less than 1 year* ***5*** *=5 years* ***9*** *=9 or more years*	*For columns H through K, enter a rating of 1 through 9, where your answer reflects your judgement about how much the outcome of interest (e.g., life expectancy in Q4 and cure rates in Q5, Q6, Q7) would increase:* ***1*** *=Not at all* ***3*** *=Slightly* ***5*** *=Moderately* ***7*** *=Considerably* ***9*** *=A great deal, doubling the outcome of interest*			
		Lower risk [8]	Higher risk [9]	Overall	Lower risk	Higher risk	Overall	Overall	Overall	Overall	Overall	Overall
		A	B	C	D	E	F	G	H	I	J	K
**Prostate**	1				3.5 (1 – 8)	7.0 (5 – 9)	6.0 (3 – 7)					
Stage I	2	9.0 (9 – 9)	8.0 (5 – 9)	8.5 (7 – 9)				7.0 (5 – 9)	3.0 (1 – 8)	3.0 (1 – 8)	3.5 (1 – 8)	3.5 (1 – 8)
Stage II	3	9.0 (8 – 9)	7.0 (5 – 9)	8.0 (7 – 9)				5.0 (4 – 7)	3.5 (2 – 6)	3.0 (2 – 6)	3.0 (2 – 6)	4.5 (2 – 6)
Stage III	4	7.0 (3 – 9)	3.5 (3 – 8)	5.5 (3 – 8)				3.0 (2 – 5)	3.0 (1 – 5)	2.5 (1 – 5)	3.0 (1 – 6)	4.5 (2 – 6)
Stage IV	5	1.0 (1 – 7)	1.0 (1 – 4)	1.0 (1 – 5)								
**Breast**	6				6.0 (2 – 8)	8.0 (5 – 9)	7.0 (4 – 8)					
Stage I	7	9.0 (8 – 9)	8.0 (6 – 9)	9.0 (7 – 9)				3.0 (2 – 9)	7.0 (3 – 8)	6.5 (3 – 8)	6.5 (3 – 8)	7.5 (3 – 9)
Stage II	8	8.0 (8 – 9)	6.5 (4 – 8)	7.5 (6 – 9)				2.0 (1 – 6)	5.5 (3 – 7)	5.5 (2 – 7)	5.5 (3 – 7)	6.0 (4 – 7)
Stage III	9	6.5 (3 – 9)	4.0 (3 – 5)	5.5 (3 – 9)				1.5 (1 – 3)	3.0 (2 – 6)	3.0 (1 – 6)	3.0 (1 – 6)	5.5 (2 – 7)
Stage IV	10	1.5 (1 – 5)	1.0 (1 – 1)	1.0 (1 – 3)								
**Lung**	11				7.0 (3 – 9)	9.0 (2 – 9)	8.0 (2 – 9)					
Stage I	12	7.5 (7 – 9)	6.0 (3 – 7)	7.0 (5 – 9)				2.0 (1 – 9)	8.0 (7 – 9)	8.0 (5 – 9)	8.0 (6 – 9)	8.5 (3 – 9)
Stage II	13	5.0 (3 – 8)	4.5 (1 – 6)	5.0 (3 – 8)				1.0 (1 – 7)	6.0 (5 – 8)	6.0 (4 – 8)	7.0 (5 – 8)	7.5 (4 – 9)
Stage III	14	3.0 (1 – 6)	2.5 (1 – 4)	3.0 (1 – 5)				1.0 (1 – 3)	3.5 (3 – 7)	3.0 (1 – 5)	4.0 (1 – 6)	5.0 (2 – 7)
Stage IV	15	1.0 (1 – 2)	1.0 (1 – 1)	1.0 (1 – 2)								

[1] Curability is the extent to which a patient diagnosed with cancer is more likely to die of something else. If a cancer is completely curable, no one will die of cancer.
[2] Includes active treatment or surveillance.
[3] For example, to answer 2G, consider the time from the beginning of stage I to the beginning of stage II. For 3G, consider the time from the beginning of stage II to the beginning of stage III. Similarly, for 4G, consider the time from the beginning of stage III to the beginning of stage IV.
[4] For stage I, assume all stage II-IV cancers are detected during stage I. For stage II, assume that all stage III and IV cancers are detected by stage II; any cancers that had been detected in stage I or II would still be detected during those stages. For the stage III, assume all stage IV cancers were diagnosed during stage III; any cancers that had been detected in stages I-III would still be detected during those stages.
[5] If there are many treatment advancements, cure rates may increase, and if treatment remains unchanged, cure rates are unlikely to change.
[6] A patient who is not cured could have increased life expectancy (e.g. still die of this cancer).
[7] Treatment consistent with NCCN guidelines.
[8] Please refer to the table above for examples of lower risk cancers.
[9] Please refer to the table above for examples of higher risk cancers.

Please enter a rating in each yellow cell, where each cell represents a cancer or cancer stage.

If there is heterogeneity within a category, do your best to imagine a **typical** patient.

Q1: What is the likelihood that patients diagnosed with this cancer at this stage today will be cured [1] with **typical treatment** [2]?

Q2: What is the likelihood that **untreated patients** with stage I or II of this cancer will progress to stage III or IV disease over their lifetime?

Q3: How long does it take this cancer to progress from the beginning of this stage [3] to the beginning of the next stage in undiagnosed patients (natural history of disease)?

Imagine an annual hypothetical screening blood test for patients ≥50 years of age that is 100% sensitive and 100% specific for the stage of cancer you are being asked to rate. Assume this test cannot differentiate between higher and lower risk cancers.

If all cancers of this type were diagnosed no later than the stage [4] indicated by the row heading, to what extent would…

Q4: …**life expectancy** increase in **all** patients with this cancer, relative to life expectancy without the screening test [6]?

Assume patients are receiving **typical treatment**.

…**cure rates** increase in **all** patients with this cancer, relative to cure rates without the screening test?

Q5: Assume patients are receiving **typical treatment**.

Q6: Assume patients are receiving **best available treatment** [7].

Now consider what medical practice might be like in 2030 [5] with cure rates higher than today's.

Q7: If all cancers of this type were diagnosed no later than this stage, to what extent would **cure rates** increase with **typical treatment** in **all** patients with this cancer, relative to 2030 cure rates without the screening test?

For columns A through F, enter a rating of 1 through 9, where:
1 *=Extremely unlikely*
3 *=Somewhat unlikely*
5 *=Neutral, neither likely nor unlikely*
7 *=Somewhat likely*
9 *=Extremely likely*

For column G, enter a rating of 1 through 9, where:
1 *=Less than 1 year*
5 *=5 years*
9 *=9 or more years*

For columns H through K, enter a rating of 1 through 9, where your answer reflects your judgement about how much the outcome of interest (e.g., life expectancy in Q4 and cure rates in Q5, Q6, Q7) would increase:
1 *=Not at all*
3 *=Slightly*
5 *=Moderately*
7 *=Considerably*
9 *=A great deal, doubling the outcome of interest*

		Q1			Q2			Q3	Q4	Q5	Q6	Q7
		Lower risk [8]	Higher risk [9]	Overall	Lower risk	Higher risk	Overall	Overall	Overall	Overall	Overall	Overall
		A	B	C	D	E	F	G	H	I	J	K
**Colon/Rectum**	16				7.0 (6 – 9)	8.0 (5 – 9)	8.0 (6 – 9)					
Stage I	17	9.0 (8 – 9)	9.0 (8 – 9)	9.0 (8 – 9)				3.5 (2 – 7)	7.5 (6 – 9)	7.0 (6 – 9)	8.0 (6 – 9)	8.0 (3 – 9)
Stage II	18	8.0 (7 – 9)	7.0 (7 – 8)	8.0 (7 – 8)				3.0 (2 – 5)	6.0 (5 – 8)	6.0 (5 – 8)	6.5 (5 – 8)	7.0 (4 – 9)
Stage III	19	6.0 (3 – 9)	5.0 (2 – 7)	5.0 (3 – 7)				1.0 (1 – 2)	5.0 (1 – 7)	4.5 (1 – 7)	5.0 (1 – 7)	6.0 (2 – 9)
Stage IV	20	1.5 (1 – 4)	1.0 (1 – 2)	1.5 (1 – 3)								
**Melanoma**	21				7.0 (4 – 9)	8.0 (6 – 9)	7.0 (6 – 9)					
Stage I	22	9.0 (8 – 9)	9.0 (7 – 9)	9.0 (8 – 9)				3.0 (1 – 5)	7.0 (2 – 9)	6.5 (2 – 9)	7.0 (2 – 9)	7.0 (2 – 9)
Stage II	23	8.0 (7 – 8)	7.0 (5 – 8)	7.0 (6 – 8)				2.0 (1 – 4)	5.0 (4 – 9)	5.0 (3 – 8)	5.0 (3 – 8)	6.0 (5 – 9)
Stage III	24	5.0 (3 – 7)	3.5 (2 – 6)	4.5 (3 – 6)				1.0 (1 – 3)	3.5 (3 – 7)	3.0 (2 – 7)	4.0 (2 – 7)	6.5 (3 – 8)
Stage IV	25	2.0 (1 – 4)	1.0 (1 – 3)	1.5 (1 – 3)								
**Kidney**	26				5.5 (4 – 9)	8.0 (4 – 9)	7.0 (5 – 9)					
Stage I	27	9.0 (8 – 9)	9.0 (4 – 9)	9.0 (7 – 9)				5.0 (1 – 7)	7.0 (3 – 9)	7.0 (5 – 9)	7.0 (5 – 9)	7.0 (3 – 9)
Stage II	28	8.0 (7 – 9)	7.0 (4 – 8)	8.0 (6 – 9)				3.0 (1 – 5)	5.5 (4 – 9)	6.0 (4 – 9)	6.0 (4 – 9)	6.5 (5 – 9)
Stage III	29	6.0 (3 – 7)	4.5 (2 – 7)	5.5 (3 – 7)				2.0 (1 – 2)	4.5 (3 – 7)	5.0 (2 – 7)	5.0 (2 – 8)	6.0 (3 – 8)
Stage IV	30	2.0 (1 – 3)	1.0 (1 – 2)	1.5 (1 – 2)								
**Head and Neck**	31				7.5 (5 – 9)	8.5 (4 – 9)	8.0 (4 – 9)					
Stage I	32	8.5 (7 – 9)	9.0 (5 – 9)	9.0 (6 – 9)				3.0 (2 – 6)	7.0 (4 – 9)	6.5 (3 – 9)	7.0 (3 – 9)	7.0 (3 – 9)
Stage II	33	8.0 (6 – 9)	7.0 (4 – 8)	7.5 (5 – 9)				2.0 (1 – 4)	5.5 (3 – 8)	5.5 (2 – 8)	6.0 (2 – 8)	6.0 (2 – 9)
Stage III	34	6.5 (3 – 9)	4.5 (3 – 7)	5.0 (3 – 8)				1.0 (1 – 2)	3.0 (2 – 7)	3.0 (1 – 7)	4.5 (1 – 7)	5.5 (1 – 8)
Stage IV	35	4.0 (1 – 8)	2.0 (1 – 6)	3.0 (1 – 8)								
**Uterus**	36				6.5 (3 – 9)	8.0 (6 – 9)	7.0 (5 – 9)					
Stage I	37	9.0 (8 – 9)	8.5 (7 – 9)	9.0 (8 – 9)				4.0 (3 – 6)	6.0 (2 – 9)	6.5 (2 – 9)	7.0 (2 – 9)	7.0 (2 – 9)
Stage II	38	8.0 (7 – 9)	7.0 (5 – 9)	8.0 (7 – 9)				3.0 (1 – 5)	5.0 (3 – 8)	5.0 (3 – 8)	5.0 (4 – 9)	6.0 (4 – 9)
Stage III	39	6.0 (5 – 9)	5.0 (4 – 8)	5.5 (5 – 8)				1.5 (1 – 3)	4.0 (3 – 7)	4.5 (2 – 7)	4.0 (2 – 7)	5.0 (3 – 8)
Stage IV	40	1.5 (1 – 4)	1.0 (1 – 2)	1.0 (1 – 3)								
**Pancreas**	41						9.0 (2 – 9)					
Stage I	42			4.0 (2 – 7)				1.0 (1 – 2)	5.5 (5 – 9)	5.0 (3 – 9)	5.5 (4 – 9)	6.5 (4 – 9)
Stage II	43			2.5 (1 – 5)				1.0 (1 – 2)	4.0 (2 – 9)	3.0 (1 – 9)	3.0 (2 – 9)	5.5 (2 – 9)
Stage III	44			1.0 (1 – 3)				1.0 (1 – 1)	2.0 (1 – 9)	1.5 (1 – 9)	2.0 (1 – 9)	3.5 (1 – 9)
Stage IV	45			1.0 (1 – 1)								

Please enter a rating in each yellow cell, where each cell represents a cancer or cancer stage.

If there is heterogeneity within a category, do your best to imagine a **typical** patient.

Q1: What is the likelihood that patients diagnosed with this cancer at this stage today will be cured [1] with **typical treatment** [2]?

Q2: What is the likelihood that **untreated patients** with stage I or II of this cancer will progress to stage III or IV disease over their lifetime?

Q3: How long does it take this cancer to progress from the beginning of this stage [3] to the beginning of the next stage in undiagnosed patients (natural history of disease)?

Imagine an annual hypothetical screening blood test for patients ≥50 years of age that is 100% sensitive and 100% specific for the stage of cancer you are being asked to rate. Assume this test cannot differentiate between higher and lower risk cancers.

If all cancers of this type were diagnosed no later than the stage [4] indicated by the row heading, to what extent would…

Q4: …**life expectancy** increase in **all** patients with this cancer, relative to life expectancy without the screening test [6]? Assume patients are receiving **typical treatment**.

…**cure rates** increase in **all** patients with this cancer, relative to cure rates without the screening test?

Q5: Assume patients are receiving **typical treatment**.

Q6: Assume patients are receiving **best available treatment** [7].

Now consider what medical practice might be like in 2030 [5] with cure rates higher than today's.

Q7: If all cancers of this type were diagnosed no later than this stage, to what extent would **cure rates** increase with **typical treatment** in **all** patients with this cancer, relative to 2030 cure rates without the screening test?

For columns A through F, enter a rating of 1 through 9, where:
1 *=Extremely unlikely*
3 *=Somewhat unlikely*
5 *=Neutral, neither likely nor unlikely*
7 *=Somewhat likely*
9 *=Extremely likely*

For column G, enter a rating of 1 through 9, where:
1 *=Less than 1 year*
5 *=5 years*
9 *=9 or more years*

For columns H through K, enter a rating of 1 through 9, where your answer reflects your judgement about how much the outcome of interest (e.g., life expectancy in Q4 and cure rates in Q5, Q6, Q7) would increase:
1 *=Not at all*
3 *=Slightly*
5 *=Moderately*
7 *=Considerably*
9 *=A great deal, doubling the outcome of interest*

		Q1			Q2			Q3	Q4	Q5	Q6	Q7
		Lower risk [8]	Higher risk [9]	Overall	Lower risk	Higher risk	Overall	Overall	Overall	Overall	Overall	Overall
		A	B	C	D	E	F	G	H	I	J	K
**Thyroid**	46				4.0 (1 – 9)	7.5 (6 – 9)	5.0 (2 – 8)					
Stage I	47	9.0 (9 – 9)	6.5 (1 – 9)	9.0 (8 – 9)				6.0 (4 – 8)	3.0 (1 – 7)	3.0 (1 – 7)	3.0 (1 – 7)	3.0 (1 – 7)
Stage II	48	9.0 (7 – 9)	5.0 (1 – 9)	8.5 (7 – 9)				5.0 (3 – 7)	3.0 (1 – 5)	3.0 (1 – 5)	3.0 (1 – 5)	3.0 (1 – 5)
Stage III	49	8.5 (4 – 9)	3.0 (1 – 9)	7.0 (4 – 9)				4.0 (2 – 5)	3.0 (2 – 5)	3.0 (1 – 5)	3.0 (1 – 5)	3.0 (2 – 6)
Stage IV	50	6.0 (1 – 9)	1.0 (1 – 5)	5.0 (1 – 7)								
**Bladder**	51						7.5 (5 – 9)					
Stage I	52			8.5 (7 – 9)				3.0 (2 – 5)	6.0 (5 – 8)	6.0 (5 – 8)	6.0 (5 – 8)	7.0 (5 – 9)
Stage II	53			6.5 (6 – 8)				2.0 (1 – 5)	5.0 (4 – 7)	4.5 (3 – 7)	5.0 (4 – 7)	6.0 (4 – 8)
Stage III	54			4.0 (3 – 5)				1.0 (1 – 2)	3.0 (2 – 7)	3.0 (2 – 5)	3.0 (2 – 5)	4.0 (3 – 8)
Stage IV	55			1.0 (1 – 2)								
**Liver/Intrahepatic Bile-duct**	56				7.5 (3 – 9)	8.0 (2 – 9)	7.5 (2 – 9)					
Stage I	57	5.0 (3 – 8)	3.5 (1 – 7)	4.0 (2 – 8)				2.0 (1 – 4)	7.0 (3 – 7)	6.0 (3 – 7)	6.5 (3 – 7)	7.0 (5 – 8)
Stage II	58	3.5 (2 – 7)	2.5 (1 – 7)	3.0 (2 – 7)				1.0 (1 – 2)	5.0 (2 – 7)	5.0 (2 – 7)	5.0 (2 – 7)	6.0 (4 – 8)
Stage III	59	2.0 (1 – 5)	1.0 (1 – 5)	1.5 (1 – 5)				1.0 (1 – 2)	2.5 (1 – 7)	2.5 (1 – 7)	2.5 (1 – 7)	4.0 (2 – 8)
Stage IV	60	1.0 (1 – 1)	1.0 (1 – 1)	1.0 (1 – 1)								
**Ovary**	61				7.0 (5 – 9)	8.0 (3 – 9)	8.0 (4 – 9)					
Stage I	62	9.0 (6 – 9)	8.0 (3 – 8)	8.0 (6 – 9)				3.0 (1 – 4)	7.5 (6 – 9)	7.0 (5 – 9)	7.0 (6 – 9)	8.0 (6 – 9)
Stage II	63	8.0 (4 – 9)	6.0 (3 – 7)	7.0 (4 – 8)				2.0 (1 – 3)	6.5 (5 – 9)	5.5 (4 – 8)	6.0 (5 – 8)	7.0 (6 – 9)
Stage III	64	6.5 (1 – 9)	2.0 (1 – 4)	3.0 (1 – 5)				1.0 (1 – 2)	3.0 (1 – 7)	3.0 (1 – 7)	3.0 (1 – 7)	5.0 (3 – 8)
Stage IV	65	4.0 (1 – 9)	1.0 (1 – 2)	1.0 (1 – 3)								
**Stomach**	66				7.5 (4 – 9)	9.0 (2 – 9)	8.0 (2 – 9)					
Stage I	67	8.0 (6 – 9)	7.0 (4 – 7)	7.0 (5 – 8)				3.0 (2 – 5)	7.5 (5 – 9)	7.0 (4 – 9)	7.0 (5 – 9)	8.0 (6 – 9)
Stage II	68	5.0 (4 – 7)	4.0 (3 – 6)	4.5 (3 – 7)				1.5 (1 – 3)	5.0 (4 – 9)	5.0 (3 – 9)	5.5 (4 – 9)	6.0 (5 – 9)
Stage III	69	3.0 (1 – 6)	2.0 (1 – 4)	2.0 (1 – 5)				1.0 (1 – 2)	3.0 (2 – 9)	3.0 (1 – 9)	3.0 (2 – 9)	4.0 (2 – 9)
Stage IV	70	1.0 (1 – 2)	1.0 (1 – 1)	1.0 (1 – 1)								
**Esophagus**	71				7.5 (4 – 9)	9.0 (2 – 9)	8.0 (2 – 9)					
Stage I	72	7.5 (4 – 9)	6.0 (4 – 7)	7.0 (4 – 8)				2.0 (2 – 5)	7.0 (5 – 9)	7.0 (4 – 9)	7.0 (5 – 9)	7.5 (6 – 9)
Stage II	73	4.5 (2 – 7)	3.5 (3 – 6)	4.0 (3 – 7)				1.0 (1 – 2)	5.0 (4 – 9)	5.0 (3 – 9)	5.0 (4 – 9)	5.5 (5 – 9)
Stage III	74	2.5 (1 – 6)	2.0 (1 – 4)	2.0 (1 – 5)				1.0 (1 – 2)	2.5 (2 – 9)	2.5 (1 – 9)	3.0 (1 – 9)	4.0 (2 – 9)
Stage IV	75	1.0 (1 – 2)	1.0 (1 – 1)	1.0 (1 – 1)								

Please enter a rating in each yellow cell, where each cell represents a cancer or cancer stage.

If there is heterogeneity within a category, do your best to imagine a **typical** patient.

Q1: What is the likelihood that patients diagnosed with this cancer at this stage today will be cured [1] with **typical treatment** [2]?

Q2: What is the likelihood that **untreated patients** with stage I or II of this cancer will progress to stage III or IV disease over their lifetime?

Q3: How long does it take this cancer to progress from the beginning of this stage [3] to the beginning of the next stage in undiagnosed patients (natural history of disease)?

Imagine an annual hypothetical screening blood test for patients ≥50 years of age that is 100% sensitive and 100% specific for the stage of cancer you are being asked to rate. Assume this test cannot differentiate between higher and lower risk cancers.

If all cancers of this type were diagnosed no later than the stage [4] indicated by the row heading, to what extent would…

Q4: …**life expectancy** increase in **all** patients with this cancer, relative to life expectancy without the screening test [6]?

Assume patients are receiving **typical treatment**.

…**cure rates** increase in **all** patients with this cancer, relative to cure rates without the screening test?

Q5: Assume patients are receiving **typical treatment**.

Q6: Assume patients are receiving **best available treatment** [7].

Now consider what medical practice might be like in 2030 [5] with cure rates higher than today's.

Q7: If all cancers of this type were diagnosed no later than this stage, to what extent would **cure rates** increase with **typical treatment** in **all** patients with this cancer, relative to 2030 cure rates without the screening test?

For columns A through F, enter a rating of 1 through 9, where:
1 *=Extremely unlikely*
3 *=Somewhat unlikely*
5 *=Neutral, neither likely nor unlikely*
7 *=Somewhat likely*
9 *=Extremely likely*

For column G, enter a rating of 1 through 9, where:
1 *=Less than 1 year*
5 *=5 years*
9 *=9 or more years*

For columns H through K, enter a rating of 1 through 9, where your answer reflects your judgement about how much the outcome of interest (e.g., life expectancy in Q4 and cure rates in Q5, Q6, Q7) would increase:
1 *=Not at all*
3 *=Slightly*
5 *=Moderately*
7 *=Considerably*
9 *=A great deal, doubling the outcome of interest*

		Q1 Lower risk [8]	Q1 Higher risk [9]	Q1 Overall	Q2 Lower risk	Q2 Higher risk	Q2 Overall	Q3 Overall	Q4 Overall	Q5 Overall	Q6 Overall	Q7 Overall
		A	B	C	D	E	F	G	H	I	J	K
**Gallbladder**	76						9.0 (2 – 9)					
Stage I	77			5.0 (4 – 8)				2.0 (1 – 3)	7.0 (4 – 9)	6.5 (4 – 9)	6.5 (5 – 9)	7.0 (5 – 9)
Stage II	78			3.0 (2 – 6)				1.0 (1 – 1)	4.5 (3 – 9)	4.5 (2 – 8)	5.0 (2 – 8)	5.0 (4 – 9)
Stage III	79			2.0 (1 – 3)				1.0 (1 – 1)	2.5 (1 – 5)	2.5 (1 – 5)	2.5 (1 – 5)	3.0 (2 – 8)
Stage IV	80			1.0 (1 – 1)								
**Cervix**	81						8.5 (6 – 9)					
Stage I	82			9.0 (8 – 9)				4.0 (1 – 5)	7.0 (5 – 9)	7.0 (5 – 9)	7.0 (6 – 9)	7.0 (5 – 9)
Stage II	83			7.0 (4 – 9)				2.5 (1 – 4)	5.0 (4 – 7)	6.0 (4 – 7)	6.5 (5 – 7)	6.5 (5 – 8)
Stage III	84			5.0 (3 – 6)				1.0 (1 – 2)	3.0 (3 – 6)	3.0 (2 – 6)	4.0 (2 – 6)	4.5 (3 – 7)
Stage IV	85			1.0 (1 – 3)								
**Sarcoma**	86				6.5 (3 – 9)	8.5 (3 – 9)	7.5 (4 – 9)					
Stage I	87	8.5 (7 – 9)	7.0 (5 – 8)	8.0 (6 – 8)				3.5 (1 – 5)	7.0 (5 – 9)	7.0 (5 – 9)	7.0 (5 – 9)	8.0 (5 – 9)
Stage II	88	7.0 (3 – 9)	6.0 (4 – 7)	6.5 (3 – 7)				2.0 (1 – 4)	5.0 (3 – 9)	5.5 (3 – 9)	6.0 (4 – 9)	6.0 (5 – 9)
Stage III	89	4.5 (1 – 6)	3.0 (1 – 4)	3.5 (1 – 5)				1.0 (1 – 2)	3.0 (2 – 7)	3.0 (2 – 8)	3.5 (2 – 8)	4.5 (3 – 9)
Stage IV	90	1.0 (1 – 3)	1.0 (1 – 1)	1.0 (1 – 2)								
**Urothelial Tract**	91						8.5 (4 – 9)					
Stage I	92			8.0 (6 – 9)				3.0 (2 – 7)	7.0 (5 – 9)	7.0 (5 – 9)	7.0 (5 – 9)	7.0 (5 – 9)
Stage II	93			5.5 (5 – 8)				2.0 (1 – 5)	5.0 (5 – 9)	5.0 (3 – 8)	5.0 (3 – 8)	5.5 (4 – 8)
Stage III	94			4.5 (3 – 5)				1.0 (1 – 3)	3.0 (2 – 7)	3.0 (2 – 7)	3.0 (2 – 7)	3.5 (3 – 8)
Stage IV	95			1.5 (1 – 3)								
**Anus**	96						8.0 (6 – 9)					
Stage I	97			9.0 (8 – 9)				3.0 (2 – 7)	5.5 (3 – 8)	5.5 (3 – 8)	6.0 (3 – 8)	7.0 (5 – 8)
Stage II	98			8.0 (7 – 8)				2.0 (1 – 5)	5.0 (3 – 7)	5.0 (3 – 8)	5.0 (3 – 8)	5.0 (5 – 8)
Stage III	99			5.5 (5 – 7)				1.0 (1 – 3)	3.5 (2 – 5)	3.0 (2 – 7)	3.5 (2 – 8)	4.0 (3 – 8)
Stage IV	100			1.0 (1 – 3)								

SECTION 2

TABLE 2. HEMATOLOGIC CANCERS: LYMPHOMA

Please enter a rating in each yellow cell, where each cell represents a cancer or cancer stage.

If there is heterogeneity within a category, do your best to imagine a **typical** patient.

Q1: What is the likelihood that patients diagnosed with this cancer at this stage today will be cured [10] with **typical treatment** [11]?

Q2: What is the likelihood that **untreated patients** with stage I or II of this cancer will progress to stage III or IV disease over their lifetime?

Q3: How long does it take this cancer to progress from the beginning of this stage [12] to the beginning of the next stage in undiagnosed patients (natural history of disease)?

Imagine an annual hypothetical screening blood test for patients ≥50 years of age that is 100% sensitive and 100% specific for the stage of cancer you are being asked to rate. Assume this test cannot differentiate between higher and lower risk cancers.

If all cancers of this type were diagnosed no later than the stage [13] indicated by the row heading, to what extent would…

Q4: …**life expectancy** increase in **all** patients with this cancer, relative to life expectancy without the screening test [15]? Assume patients are receiving **typical treatment**.

…**cure rates** increase in **all** patients with this cancer, relative to cure rates without the screening test?

Q5: Assume patients are receiving **typical treatment**.

Q6: Assume patients are receiving **best available treatment** [16].

Now consider what medical practice might be like in 2030 [14] with cure rates higher than today's.

Q7: If all cancers of this type were diagnosed no later than this stage, to what extent would **cure rates** increase with **typical treatment** in **all** patients with this cancer, relative to 2030 cure rates without the screening test?

For columns A through F, enter a rating of 1 through 9, where:
1 *=Extremely unlikely*
3 *=Somewhat unlikely*
5 *=Neutral, neither likely nor unlikely*
7 *=Somewhat likely*
9 *=Extremely likely*

For column G, enter a rating of 1 through 9, where:
1 *=Less than 1 year*
5 *=5 years*
9 *=9 or more years*

For columns H through K, enter a rating of 1 through 9, where your answer reflects your judgement about how much the outcome of interest (e.g., life expectancy in Q4 and cure rates in Q5, Q6, Q7) would increase:
1 *=Not at all*
3 *=Slightly*
5 *=Moderately*
7 *=Considerably*
9 *=A great deal, doubling the outcome of interest*

		Q1			Q2			Q3	Q4	Q5	Q6	Q7
		Lower risk [17]	Higher risk [18]	Overall	Lower risk	Higher risk	Overall	Overall	Overall	Overall	Overall	Overall
		A	B	C	D	E	F	G	H	I	J	K
**Lymphoma**	101				5.0 (3 – 9)	9.0 (4 – 9)	7.0 (6 – 9)					
Stage I	102	9.0 (7 – 9)	8.0 (5 – 9)	8.0 (7 – 9)				4.0 (2 – 7)	4.5 (2 – 8)	4.0 (1 – 9)	5.0 (2 – 9)	7.0 (3 – 9)
Stage II	103	8.0 (6 – 9)	7.0 (3 – 8)	8.0 (5 – 8)				2.5 (1 – 5)	4.0 (2 – 8)	4.0 (2 – 9)	4.5 (2 – 9)	6.0 (3 – 9)
Stage III	104	7.0 (3 – 9)	5.5 (1 – 7)	6.0 (2 – 8)				2.0 (1 – 3)	3.0 (1 – 7)	3.0 (1 – 8)	3.5 (1 – 8)	5.0 (3 – 9)
Stage IV	105	6.0 (2 – 9)	4.5 (1 – 6)	5.0 (1 – 6)								

[10] Curability is the extent to which a patient diagnosed with cancer is more likely to die of something else. If a cancer is completely curable, no one will die of cancer.
[11] Includes active treatment or surveillance.
[12] For example, to answer 2G, consider the time from the beginning of stage I to the beginning of stage II. For 3G, consider the time from the beginning of stage II to the beginning of stage III. Similarly, for 4G, consider the time from the beginning of stage III to the beginning of stage IV.
[13] For stage I, assume all stage II-IV cancers are detected during stage I. For stage II, assume that all stage III and IV cancers are detected by stage II; any cancers that had been detected in stage I or II would still be detected during those stages. For the stage III, assume all stage IV cancers were diagnosed during stage III; any cancers that had been detected in stages I-III would still be detected during those stages.
[14] If there are many treatment advancements, cure rates may increase, and if treatment remains unchanged, cure rates are unlikely to change.
[15] A patient who is not cured could have increased life expectancy (e.g. still die of this cancer).
[16] Treatment consistent with NCCN guidelines.
[17] Please refer to the table above for examples of lower risk cancers.
[18] Please refer to the table above for examples of higher risk cancers.

TABLE 3. OTHER HEMATOLOGIC CANCERS

Please enter a rating in each yellow cell, where each cell represents a cancer type. If there is heterogeneity within a category, do your best to imagine a **typical** patient.		Q1: What is the likelihood that patients diagnosed with this cancer today will be cured [19] with **typical treatment** [20]?			Q2: What is the likelihood that **untreated patients** early [21] in their disease course with this cancer will progress and require treatment?			Imagine an annual hypothetical screening blood test for patients ≥50 years of age that is 100% sensitive and 100% specific for the cancer you are being asked to rate. Assume this test cannot differentiate between higher and lower risk cancers. If all cancers of this type were diagnosed earlier [21] in their disease course, to what extent would…			
								Q3: …**life expectancy** increase in **all** patients with this cancer, relative to life expectancy without the screening test [23]? Assume patients are receiving **typical treatment**.	…**cure rates** increase in **all** patients with this cancer, relative to cure rates without the screening test? Q4: Assume patients are receiving **typical treatment**.	Q5: Assume patients are receiving **best available treatment** [24].	Now consider what medical practice might be like in 2030 [22] with cure rates higher than today's. Q6: If all cancers of this type were diagnosed earlier in their disease course, to what extent would **cure rates** increase with **typical treatment** in **all** patients with this cancer, relative to 2030 cure rates without the screening test?
		For columns A through F, enter a rating of 1 through 9, where:<br>**1** *=Extremely unlikely*<br>**3** *=Somewhat unlikely*<br>**5** *=Neutral, neither likely nor unlikely*<br>**7** *=Somewhat likely*<br>**9** *=Extremely likely*						*For columns G through J, enter a rating of 1 through 9, where your answer reflects your judgement about how much the outcome of interest (e.g., life expectancy in Q3 and cure rates in Q4, Q5, Q6) would increase:*<br>**1** *=Not at all*<br>**3** *=Slightly*<br>**5** *=Moderately*<br>**7** *=Considerably*<br>**9** *=A great deal, doubling the outcome of interest*			
		Lower risk [25]	Higher risk [26]	Overall	Lower risk	Higher risk	Overall	Overall	Overall	Overall	Overall
		A	B	C	D	E	F	G	H	I	J
ALL [27]	106			5.0 (2 – 8)			9.0 (1 – 9)	3.5 (1 – 7)	3.0 (1 – 7)	3.5 (1 – 7)	5.0 (1 – 8)
CLL [28]	107	5.0 (1 – 9)	2.5 (1 – 7)	4.0 (1 – 8)	3.5 (1 – 7)	7.0 (1 – 9)	5.0 (1 – 8)	3.0 (1 – 5)	3.0 (1 – 5)	3.0 (1 – 6)	4.5 (1 – 7)
**Lymphoid Leukemia**	108			5.5 (3 – 7)			7.0 (3 – 9)	3 (1 – 6)	3 (1 – 7)	3.5 (1 – 7)	5 (1 – 7)
**Plasma Cell Neoplasm** (Multiple Myeloma)	109	7.0 (4 – 9)	2.5 (1 – 5)	3.5 (2 – 5)	6.0 (2 – 7)	8.0 (6 – 9)	7.0 (6 – 8)	4.5 (3 – 6)	3.5 (2 – 6)	4.5 (2 – 6)	5.0 (3 – 7)
AML	110	6.0 (3 – 7)	2.5 (2 – 4)	3.5 (3 – 5)	8.5 (7 – 9)	9.0 (7 – 9)	9.0 (7 – 9)	4.5 (1 – 7)	4.0 (1 – 7)	5.0 (1 – 7)	5.0 (1 – 8)
CML	111			7.0 (5 – 8)			8.0 (6 – 9)	3.0 (1 – 7)	2.0 (1 – 7)	3.0 (1 – 7)	4.0 (1 – 8)
**Myeloid Neoplasm**	112			4.0 (3 – 7)			8.0 (7 – 9)	4.0 (1 – 7)	3.0 (1 – 7)	3.5 (1 – 7)	5.0 (1 – 8)

[19] Curability is the extent to which a patient diagnosed with cancer is more likely to die of something else. If a cancer is completely curable, no one will die of cancer.
[20] Includes active treatment or surveillance.
[21] We define "early" as asymptomatic but already meeting the definition of cancer (e.g., not having a precursor condition).
[22] If there are many treatment advancements, cure rates may increase, and if treatment remains unchanged, cure rates are unlikely to change.
[23] A patient who is not cured could have increased life expectancy (e.g. still die of this cancer).
[24] Treatment consistent with NCCN guidelines.
[25] Please refer to the table above for examples of lower risk cancers.
[26] Please refer to the table above for examples of higher risk cancers.
[27] Acute lymphocytic (or lymphoblastic) leukemia.
[28] Chronic lymphocytic (or lymphoblastic) leukemia.
[29] Acute myeloid leukemia.
[30] Chronic myeloid leukemia.
