# Supplementary material for: Impact of early detection on cancer curability: A modified Delphi panel study
Source: PLoS One. 2022 Dec 21;17(12):e0279227. doi: 10.1371/journal.pone.0279227 (PMC9770338; doi:10.1371/journal.pone.0279227)
Supplement: Attachment — Submitted filename: Response to Reviewers.docx [file pone.0279227.s005.docx]

GRAIL’s response to reviewers’ comments (see below in red):

Thank you for your review and comments. Based on the feedback provided, we have made edits to the manuscript and have included our responses below to the reviewers’ comments. The new changes have been identified in the revised manuscript.

Journal Requirements:

Please review your reference list to ensure that it is complete and correct. If you have cited papers that have been retracted, please include the rationale for doing so in the manuscript text, or remove these references and replace them with relevant current references. Any changes to the reference list should be mentioned in the rebuttal letter that accompanies your revised manuscript. If you need to cite a retracted article, indicate the article’s retracted status in the References list and also include a citation and full reference for the retraction notice.

**Response**: No changes need to be made to the reference list.

Additional Editor Comments (if provided):

Thank you for this interesting and conceptual study. There is no doubt that early diagnosis and prompt treatment remains our main motto. However, before we jump into universal screening for all cancers, we need to know the limitations and practical aspects of it. We certainly need to weigh benefit / risk of screening to provide comprehensive benefit to patients. This study is an initial step in this endeavor. As highlighted in the reviews, one of the main limitations of this study is incorporating opinion of an Oncologist on all cancers. I hope this will be mentioned in the limitations. I believe this study will invite further dedicated studies with more concrete data from experts in each type of cancer by implementing real data from the institutional experience and comparing with reference information such as from the SEER.

**Response**: Thank you for your comments. We have included as a limitation that the panel of experts did include one general oncologist, though we have also further clarified that the rest of the panel did represent the majority of cancer types covered within this study in lines 322-324 of the discussion section.

[Note: HTML markup is below. Please do not edit.]

Reviewers' comments:

Reviewer's Responses to Questions

**Comments to the Author**

1. If the authors have adequately addressed your comments raised in a previous round of review and you feel that this manuscript is now acceptable for publication, you may indicate that here to bypass the “Comments to the Author” section, enter your conflict of interest statement in the “Confidential to Editor” section, and submit your "Accept" recommendation.

Reviewer #1: (No Response)

2. Is the manuscript technically sound, and do the data support the conclusions?

The manuscript must describe a technically sound piece of scientific research with data that supports the conclusions. Experiments must have been conducted rigorously, with appropriate controls, replication, and sample sizes. The conclusions must be drawn appropriately based on the data presented.

Reviewer #1: Yes

3. Has the statistical analysis been performed appropriately and rigorously?

Reviewer #1: Yes

4. Have the authors made all data underlying the findings in their manuscript fully available?

The [PLOS Data policy](http://www.plosone.org/static/policies.action#sharing) requires authors to make all data underlying the findings described in their manuscript fully available without restriction, with rare exception (please refer to the Data Availability Statement in the manuscript PDF file). The data should be provided as part of the manuscript or its supporting information, or deposited to a public repository. For example, in addition to summary statistics, the data points behind means, medians and variance measures should be available. If there are restrictions on publicly sharing data—e.g. participant privacy or use of data from a third party—those must be specified.

Reviewer #1: Yes

5. Is the manuscript presented in an intelligible fashion and written in standard English?

PLOS ONE does not copyedit accepted manuscripts, so the language in submitted articles must be clear, correct, and unambiguous. Any typographical or grammatical errors should be corrected at revision, so please note any specific errors here.

Reviewer #1: Yes

6. Review Comments to the Author

Please use the space provided to explain your answers to the questions above. You may also include additional comments for the author, including concerns about dual publication, research ethics, or publication ethics. (Please upload your review as an attachment if it exceeds 20,000 characters)

Reviewer #1: The Authors explained in their response letter that panelists were explicit on whether they had any
experience with a certain cancer type or not. This information is important for the interpretation of the findings and shall be added to the manuscript: please include the number of experts with explicit experience for each specific cancer type.
In addition, please clarify whether scores were collected from all experts for all cancer types? Or for each cancer type, scores were collected only from experts with declared experience with the particular cancer type?

**Response**: Thank you for these points. While we had one general practice oncologist, the remaining specialists specified their expertise within the following cancer types: hematologic (2), prostate (1), breast (3), lung (2), colorectal (2), gastrointestinal, including, but not limited to, bile duct, pancreas, esophageal, stomach (1), head and neck (1), liver (1), gynecological, including, but not limited to, cervical and ovarian (1); sarcoma (2). The scores were collected for all cancer types from all experts. As expected in the Delphi approach, the discussion did include the group hearing from experts with more experience for certain cancer types and then each panelist synthesizing that information with their own experience in their individual ratings. We addressed these two points within the methods section in lines 112-115 and 136.

7. PLOS authors have the option to publish the peer review history of their article ([what does this mean?](https://journals.plos.org/plosone/s/editorial-and-peer-review-process#loc-peer-review-history)). If published, this will include your full peer review and any attached files.


If you choose “no”, your identity will remain anonymous but your review may still be made public.


**Do you want your identity to be public for this peer review?** For information about this choice, including consent withdrawal, please see our [Privacy Policy](https://www.plos.org/privacy-policy).

Reviewer #1: **Yes:** Janos G. Pitter MD, PhD

[NOTE: If reviewer comments were submitted as an attachment file, they will be attached to this email and accessible via the submission site. Please log into your account, locate the manuscript record, and check for the action link "View Attachments". If this link does not appear, there are no attachment files.]

While revising your submission, please upload your figure files to the Preflight Analysis and Conversion Engine (PACE) digital diagnostic tool, <https://pacev2.apexcovantage.com/>. PACE helps ensure that figures meet PLOS requirements. To use PACE, you must first register as a user. Registration is free. Then, login and navigate to the UPLOAD tab, where you will find detailed instructions on how to use the tool. If you encounter any issues or have any questions when using PACE, please email PLOS at [figures@plos.org](mailto:figures@plos.org). Please note that Supporting Information files do not need this step.

**Response**: The figure files have been uploaded to PACE.
